# Gastric cancer cell-derived exosomal miRNA-128-3p promotes angiogenesis by targeting SASH1

**DOI:** 10.3389/fonc.2024.1440996

**Published:** 2024-11-27

**Authors:** Hao Yan, Xinyu Cai, Jianna Zhang, Hongpeng Zhao, Hongwen Wu, Jingbo Zhang, Lanzhi Xu, Shizheng Liu, Yuanwei Zang, Shanshan Fu

**Affiliations:** ^1^ Department of Gastroenterology, Shandong Public Health Clinical Center, Shandong University, Jinan, China; ^2^ Department of Gastroenterology, The First Affiliated Hospital of Shandong First Medical University & Shandong Provincial Qianfoshan Hospital, Jinan, Shandong, China; ^3^ Shandong First Medical University & Shandong Academy of Medical Sciences, Jinan, Shandong, China; ^4^ Department of Gastrointestinal Surgery, The First Affiliated Hospital of Shandong First Medical University & Shandong Provincial Qianfoshan Hospital, Jinan, Shandong, China; ^5^ Department of Urology, Qilu Hospital, Cheeloo College of Medicine, Shandong University, Jinan, China

**Keywords:** gastric cancer, exosome, miR-128-3p, targeted therapy, angiogenesis

## Abstract

Exosomes, key components of the tumour microenvironment, can mediate intercellular communication through the delivery of various signalling molecules, including microribonucleic acids (miRNAs), and ultimately participate in regulating the process of tumour development. In this study, we aimed to investigate the reason and mechanism by which exosomal miRNAs derived from gastric cancer cells affect carcinogenesis and metastasis. Among these miRNAs, microRNA-128-3p (miR-128-3p) was highly expressed in serum exosomes isolated from gastric cancer patients, as confirmed by high-throughput sequencing and subsequent experiments. Coculture of gastric cancer-derived exosomes overexpressing miR-128-3p with human umbilical vein endothelial cells (HUVECs) significantly enhanced HUVEC proliferation, migratio n and angiogenesis. Bioinformatics analysis suggested SASH1 as the target gene of miR-128-3p. The dual luciferase assay and Western blot analysis results confirmed that miR-128-3p directly targeted SASH1 to inhibit its expression in HUVECs. Therefore, this study provides preliminary evidence that gastric cancer-derived exosomal miR-128-3p promotes tumour angiogenesis by targeting SASH1, reveals the potential diagnostic and therapeutic value of cancer-derived exosomal miR-128-3p, and provides new insights into the novel molecular mechanisms regulating metastasis. This study provides further information for understanding the role of gastric cancer-derived exosomal miR-128-3p in cancer progression and to discover new therapeutic targets.

## 1 Introduction

Gastric cancer (GC) is the fifth most common malignancy and the third most common cause of cancer-related death worldwide (1). In recent years, surgical treatment, endoscopic treatment, chemotherapy, radiotherapy, and targeted therapy have made great progress in the treatment of GC (2). The main treatment for early gastric cancer is endoscopic resection, and the 5-year survival rate after surgery can reach more than 90%; non-early operable gastric cancer is treated with surgery, and the addition of adjuvant chemotherapy can increase the 5-year survival rate of patients with stage II and III cancer from 23% to 36%. However, advanced gastric cancer is mainly treated with sequential chemotherapy, with a median survival of less than 1 year (1, 3, 4).

Angiogenesis is a complex series of developmental processes leading to the generation of new capillaries based on existing capillaries through a process predominantly involving sprouting. The basic steps include proteinase production, matrix degradation, endothelial cell proliferation and migration, formation of new tubular vessel branches, anastomosis of newly generated vessels, and synthesis of a new basement membrane. Angiogenesis is not only widely involved in various physiopathological phenomena in humans but is also a key factor in various stages of tumorigenesis, progression, invasion and metastasis (5). As early as the 1970s, Folkman et al. proposed the concept of anti-angiogenic therapy (6). This group was the first to advocate the hypothesis that “tumour growth is dependent on angiogenesis”, a relationship that was later confirmed as a hallmark of cancer (7). During tumour development, angiogenesis can provide tumour tissues with essential oxygen and nutrients and support the removal of metabolic waste, promoting tumour development.Tumour neovascularization is often structurally and functionally underdeveloped, and the vessel wall is prone to leakage, resulting in the ability of tumour cells to directly enter the vasculature and undergo distant metastasis via the systemic blood circulation. Moreover, neovascularization can promote the uptake of required nutrients by tumours and accelerate their growth (8). Therefore, angiogenesis is the basis of tumour development, and by inhibiting this process, tumour progression can be inhibited. In the current era of tumour treatment, antiangiogenic therapy has achieved remarkable results in a variety of cancers (such as gastric, lung, colorectal and breast cancers) (9). The molecular mechanisms of tumour angiogenesis have also received increasing attention in the study of gastric cancer.

The tumour microenvironment (TME) plays an essential role in tumour biology. We have recognized that the dynamic exchange of information between cancer cells and the surrounding microenvironment is a fundamental factor that allows tumors to develop, evolve and metastasize (10). In addition to direct cell-to-cell contact, intercellular communication via secreted factors plays a key role in intercellular signaling. With the continuous exploration of TME in recent decades, the process of exosome-mediated information exchange between tumor cells and the surrounding microenvironment has become a hot topic for mechanistic studies.

Exosome (Exos) refers to a specific type of small extracellular vesicle, as outlined in the MISEV guidelines (MISEV2018, MISEV2023). Exosomes are nanoscale vesicles (30-150 nm in diameter) that are actively secreted by cells and stably present in various biological fluids (such as blood, urine, and saliva) and in cell culture medium (11). In addition, exosomes can reflect the phenotypic characteristics of their source cells to some extent (12). Furthermore, exosomes can package and transport a variety of bioactive contents, including proteins, DNA, messenger ribonucleic acids (mRNAs) and microRNAs (miRNAs), between cells (13). It has been shown that increased production of exosomes in the TME can influence tumour development in various cancers (14). Exosomes and their transported cargo are involved in regulating several developmental processes of GC, such as angiogenesis, immune escape, invasion, metastasis, and chemoresistance, by participating in the exchange of information (15). On the one hand, tumour cell-derived exosomes transport a variety of signalling molecules including miRNAs to synergistically regulate angiogenesis, thereby affecting gastric cancer progression. Identifying and understanding the mechanism of action of these bioactive factors is important for discovering therapeutic targets in advanced gastric cancer. On the other hand, there is continuous evidence of the feasibility of exosomes as novel delivery vehicles for therapeutic applications. However, the diversity of contents transported by exosomes makes it difficult to standardise the preparation of exosomes as therapeutic vehicles. Therefore, identifying and exploring the bioactive molecules carried by these exosomes not only helps to understand the mechanism of action of exosomes *in vivo*, but also facilitates the preparation of engineered exosomes.

Along with the rapid development of molecular biology, many noncoding RNAs, such as miRNAs, long noncoding RNAs (lncRNAs), and circular RNAs (circRNAs), have been highlighted. miRNAs, one of the most important contents of exosomes, are small, noncoding single-stranded RNAs containing approximately 18-24 nucleotides. MiRNAs negatively regulate gene expression by binding to the 3’-untranslated region (3’ UTR) of target genes to degrade or block the translation of the target mRNAs (16). Thus, miRNAs can target and regulate multiple genes involved in the occurrence and progression of tumours (17), and regulating the expression levels of miRNAs can promote or suppress tumour progression (18).

Exosomal miRNAs can exist more stably in body fluids than free miRNAs due to the lipid bilayer structure of exosomes, which prevents their degradation by RNases and other enzymes (19). It has also been shown that exosomal miRNAs are involved in promoting gastric cancer angiogenesis and influence the development of gastric cancer (20). The aim of this study was to demonstrate the effect of gastric cancer-derived exosomal miR-128-3p on tumour angiogenesis and determine the related molecular mechanism.

In this study, after performing high-throughput sequencing of exosomes isolated from serum samples of GC patients and healthy individuals, we combined functional assays and quantitative real-time PCR (qRT−PCR) data to identify miR-128-3p, which was significantly upregulated in GC patient serum-derived exosomes. By Kyoto Encyclopedia of Genes and Genomes (KEGG) pathway analysis, we found that miR-128-3p may be involved in angiogenesis. By coculturing HUVECs with GC-derived exosomes overexpressing miR-128-3p, we found that gastric cancer-derived exosomal miR-128-3p facilitates tumour angiogenesis. In addition, we demonstrated that miR-128-3p acts on the target gene SAM and SH3 domain-containing 1 (SASH1), thus providing a new potential target for anti-angiogenic therapy of GC.

## 2 Materials and methods

### 2.1 Clinical specimens

All serum samples were obtained from 13 patients with pathologically confirmed gastric cancer and 13 healthy individuals at the First Affiliated Hospital of Shandong First Medical University. Serum samples from 3 GC patients and 3 healthy individuals were subjected to gene sequencing, and serum samples from 10 GC patients and 10 healthy individuals were used for qRT−PCR. Informed consent was obtained from all subjects. The study was approved by the institutional review board of the First Affiliated Hospital of Shandong First Medical University (Ethics approval No. YXLL-KY-2022039). Research was conducted in accordance with the tenets of the Declaration of Helsinki.

### 2.2 Cell culture

AGS cells (AGSs), a GC cell line, were obtained from the Chinese Academy of Sciences (Shanghai, China) and cultured in Dulbecco’s modified Eagle’s Medium (DMEM; Gibco, USA) supplemented with 10% foetal bovine serum (FBS; Gibco, USA). Human umbilical vein endothelial cells (HUVECs) were purchased from ScienCell and cultured in endothelial cell medium (ECM; ScienCell, USA). All cells were cultured in an incubator at 37 °C in 5% CO_2_. The AGS cell line was infected with a lentivirus overexpressing hsa-miR-128-3p and a control (NC) lentivirus, respectively. After culturing the cells in serum-free of exosomes, the supernatants of the two groups of cells were collected. Exosomes from both groups were extracted by ultracentrifugation and named NC-Exo and miR128-3p-Exo, respectively. The total protein concentration of the exosomes was determined using the BCA assay. HUVEC cells were treated with 100 ug/ml of exosomes for 24 hours, after which corresponding cell function tests and Western Blot assays were performed.

### 2.3 Exosome isolation

Exosomes were isolated from serum samples and cell lines by ultracentrifugation. In brief, after cells and other debris were removed by centrifugation at 2000×g for 15 minutes and 10000×g for 30 minutes, the supernatant was centrifuged at 120,000×g for 70 minutes. Then, the pellet was resuspended in PBS and centrifuged at 120,000×g for 70 minutes. Finally, exosomes were collected from the pellet and resuspended in PBS.

### 2.4 Exosome characterization

The morphology of the collected exosomes was examined using a transmission electron microscope (TEM; HT7800, Hitachi, Japan). The size distribution of the collected exosomes was determined using a nanoparticle tracking analysis instrument (ZetaView PMX110, Particle Metrix). In addition, HSP70 (66183-1-Ig, Proteintech, 1:2000), TSG101 (14497-1-AP, Proteintech, 1:2000), and CD81 (66866-1-Ig, Proteintech, 1:2000) were used as markers for exosomes and detected by Western blot (WB) analysis.

### 2.5 High-throughput sequencing

RNA extraction from exosomes isolated from serum samples of 3 GC patients and 3 healthy individuals, miRNA library preparation, sequencing and data analysis were performed by Beijing Genomics Institute (Beijing, China).

### 2.6 KEGG pathway analysis

The targets of the 30 most abundant miRNAs were determined using the RNAhybrid, miRanda and TargetScan databases. We selected the overlapping targets predicted by all three databases as the candidate target genes of the miRNAs. Then, biological pathway analysis was performed based on the KEGG biological pathway database.

### 2.7 qRT−PCR

Quantitative real-time PCR (qRT−PCR) was applied to validate the sequencing results in 10 GC patients and 10 healthy individuals as well as the lentiviral transduction efficiency. Exosomes were isolated from serum or AGS cell supernatant, and total RNA was then extracted from exosomes with TRIzol reagent (Invitrogen, USA). Following the manufacturer’s protocol, miRNAs were reverse transcribed to cDNA using the miRNA reverse transcription kit, and miRNA fluorescence quantification was performed using SYBR Green Mix. The relative expression levels of miRNAs were calculated by the 2^-ΔΔCt^ method, which indicates the relative expression level of the target gene in each experimental sample compared with the control sample, i.e., ΔΔCt = (ΔCt sample - ΔCt control). U6 was used as an internal control for miRNAs, and the primer sequences were as follows:

5’-GTGCTCGCTTCGGCAGCACATAT-3’ (U6, sense);

5’-AGTGCAGGGTCCGAGGTATT-3’ (U6, antisense);

5’-CGCGTCACAGTGAACCGGT-3’ (miRNA128-3p, sense);

5’- AGTGCAGGGTCCGAGGTATT -3’ (miRNA128-3p, antisense).

### 2.8 Exosome uptake assay

When AGS cells were cultured to 70% confluence, the medium was replaced with exosome-free medium. After 48 h, the cell supernatant was collected, and exosomes were isolated by ultracentrifugation. The exosomes were labelled with the green fluorescent dye PKH67 (Sigma, USA), sterilized by passage through a 0.22 µm filter membrane and incubated with HUVECs. After incubation for 0 h, 4 h, 8 h and 16 h, the uptake of exosomes was examined by fluorescence microscopy (Olympus, Japan). Prior to imaging, cells were washed with PBS and stained with DAPI.

### 2.9 Lentiviral transduction

The miR-128-3p overexpression lentivirus was purchased from Oligobio (Beijing, China), and the lentiviral overexpression backbone plasmid was pCDH-CMV-MCS-EF1-copGFP-T2A-Puro, Amp+. Empty lentiviral vectors served as negative controls (NCs). AGS cells were infected with the miR-128-3p overexpression lentivirus and NC lentivirus in the presence of 10 μg/mL polybrene (Sigma−Aldrich).

### 2.10 Cell proliferation assay

HUVECs were treated with miR-128-3p-rich exosomes (miR-128-3p-Exos, negative control exosomes (NC-Exos) or culture medium only (CON). HUVECs were then harvested and seeded in triplicate in a 96-well plate. Cell Counting Kit-8 (CCK8; MCE, HY K0301) solution (5 mg/mL, 10 μL per well) was added, and the cells were continuously cultured for 2 h at 37°C. The absorbance at 450 nm was measured on an automated microplate reader (Tecan F50) at different time points (0 h, 24 h, 48 h, 72 h, and 96 h post-incubation).

### 2.11 Transwell assay

HUVECs were treated with miR-128-3p-Exos, NC-Exos or CON. Then, 1.5× $10^{5}$ HUVECs in serum-free culture medium were transferred into the upper chamber. Medium containing 30% FBS was added to the lower chamber as a chemoattractant. After 24 h of incubation, nonmigrated cells on the upper surface of the inner membrane were removed, and cells that migrated to the bottom of the membrane were fixed and stained. Images (200×) were acquired under an inverted microscope (Olympus, CKX-51), and the migrated cells were counted using ImageJ software.

### 2.12 Wound healing assay

HUVECs were treated with miR-128-3p-Exos, NC-Exos or CON. Then, HUVECs were seeded in 24-well plates at a density of 1.2× $10^{6}$ cells/well. When the cells formed a monolayer, a wound was generated by scratching the monolayer using a sterile 10 µL pipette tip. The floating cells were removed by washing with PBS, and serum-free medium was then added for culture in a 5% CO_2_ incubator at 37°C. Images (40×) were acquired with an inverted microscope (Olympus, CKX-51) at 0 h and 24 h after scratching.

### 2.13 Matrigel tube formation assay

The Matrigel tube formation assay was performed as previously described (21), and the specific steps were performed according to the Corning Matrigel instructions. In brief, HUVECs were treated with miR-128-3p-Exos, NC-Exos or CON. Then, HUVECs were collected and seeded in 24-well plates precoated with Matrigel at a density of 7× $10^{5}$ cells/well. Then, the plates were incubated at 37°C for 6 h to 8 h. Images (40×) were acquired under an inverted microscope (Olympus, CKX-51) to analyse differences in tube formation.

### 2.14 Luciferase activity assay

Experimentally relevant miRNA analogues (mimics) were synthesized by Beijing Olinger Biotechnology Co. The 3’ UTR fragment of the SASH1 gene containing the wild-type or mutant miR-128-3p binding site was amplified by PCR and inserted into the psiCHECKTM-2 vector (Promega,USA). HEK293T cells were cotransfected with the wild-type or mutant SASH1-3’ UTR vector using Lipofectamine 2000 reagent (Invitrogen,USA). Then, the luciferase activity assay was performed with the Dual-Luciferase Reporter Assay System (Promega, E1910).

### 2.15 miRNA target prediction

Prediction of hsa-miR-128-3p target genes was performed using all 12 databases/methods provided by miRWalk2.0 (DIANA-microTv4.0, DIANA-microT-CDS, miRanda-rel2010, mirBridge, miRDB4.0, miRmap, miRNAMap, doRiNA i.e., PicTar2, PITA RNA22v2, RNAhybrid2.1, and TargetScan 6.2). Results supported by more than half (>6) of the databases/methods were considered reliable results for subsequent analysis. Currently known tumour suppressor genes were obtained from the TSGene database (v2.0), and gastric cancer-related causative genes were obtained from the DisGeNET and Phenopedia gene banks. All results obtained from the above analyses were cross-analysed using Jveen, and the results obtained from the correlated intersections were considered hsa-miR-128-3p-regulated oncogenic target genes in gastric cancer.

### 2.16 Western blotting

The expression of exosome marker proteins was measured by Western blot analysis: CD81, HSP70 and TSG101 were exosome positive marker proteins. Cryopreserved exosomes were slowly thawed on ice, and according to the exosome protein concentration detected by the BCA kit, each exosome concentration was adjusted using D-PBS buffer, and an appropriate amount of exosomes was taken into a new 1.5-ml centrifuge tube, 1/4 exosome volume of 5xSDS-PAGE protein loading buffer and 1/100 exosome volume of PMSF were added, mixed by slow blowing using a pipette, and denatured in a metal bath for 10 minutes to label the relevant information.

The expression of SASH1 was measured by Western blot analysis: HUVECs were transfected with miR-128-3p mimics or NC mimics, and a blank control group was also established. Proteins in the samples were separated by 10% SDS−PAGE and transferred onto a PVDF membrane. The antibodies used in this study were as follows: anti-SASH1 (ABclonal, A15248, 1:1000) and anti-GAPDH (Proteintech, 60004-1-Ig, 1:5000) primary antibodies, and secondary antibodies (ABclonal, AS014, and AS003, 1:5000). Bands were detected using an iBright FL1500 imaging system (Invitrogen), and band densities were quantified using ImageJ software.

### 2.17 Statistical analysis

All data were statistically analysed using GraphPad Prism version 9.0.0 (GraphPad Software, San Diego, CA). Data were obtained from three experiments and are expressed as the means ± standard deviations. The significance of differences between groups was determined using an unpaired t test. Asterisks indicate the level of significance between the conditions for each group. The *p* values are marked as follows: *P<0.05, **P<0.01, and ***P<0.001.

## 3 Result

### 3.1 Characterization of purified exosomes in serum

**Figure 1:**
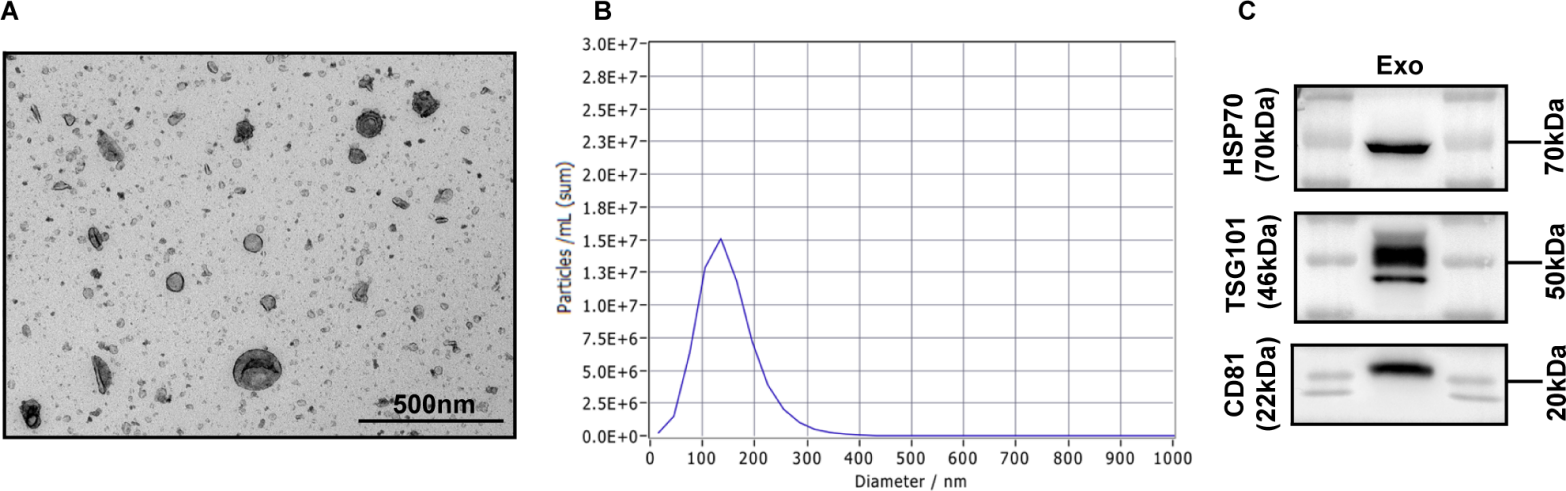
Characterization of exosomes. **(A)** Morphology observed by transmission electron microscopy. Scale bar = 500 nm. **(B)** Particle size distribution measured by nanoparticle tracking analysis. **(C)** The exosomal markers HSP70, TSG101 and CD81 were detected by Western blot analysis.

We isolated exosomes from the serum of GC patients and healthy individuals and the culture supernatants of gastric cancer cells (AGS). The vesicles had a characteristic ‘saucer’ shape as visualized by electron microscopy (**Figure 1A**), consistent with the expected appearance of exosomes. Next, we demonstrated by NanoSight tracking analysis that the isolated exosomes had an average size of 30-200 nm, consistent with the reported size of exosomes (**Figure 1B**). In addition, Western blot analysis revealed that the isolated exosomes expressed the widely accepted exosomal markers HSP70, TSG101, and CD81 (**Figure 1C**). The results of the above experiments indicated that we successfully extracted exosomes, which could be used for subsequent experiments.

### 3.2 Dysregulated miRNAs in serum-derived exosomes from gastric cancer patients

**Figure 2:**
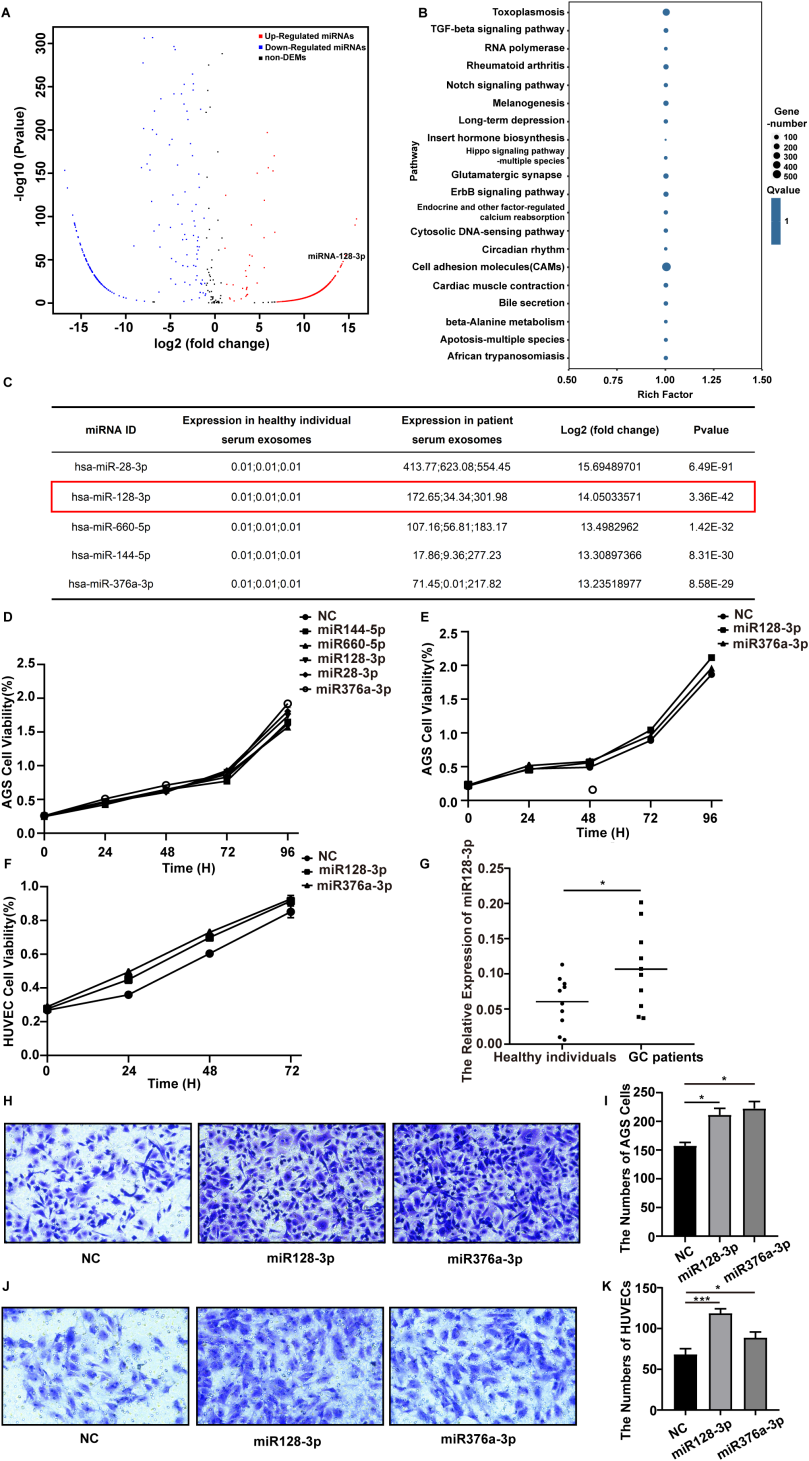
Differentially expressed miRNAs in serum-derived exosomes isolated from GC patients were selected. **(A)** Volcano plot of differentially expressed miRNAs in serum-derived exosomes from GC patients and healthy individuals based on analysis of the high-throughput sequencing data. The red dots represent upregulated miRNAs, and the blue dots represent downregulated miRNAs. |FC| > 2.0 and p value <0.05 were used as the screening criteria. **(B)** KEGG pathway analysis of the targets of the 30 most abundant miRNAs in exosomes. **(C)** Detailed information on the significantly upregulated miRNAs is listed. **(D–F)** The proliferation ability of AGS cells and HUVECs after transfection with different miRNAs was assessed using a CCK8 assay, and the proliferation curve was plotted. **(G)** qRT-PCR analysis of miR-128-3p expression in serum-derived exosomes from GC patients and healthy individuals (*P<0.05). **(H, I)** Transwell assays were performed to assess the migration ability of AGS cells after transfection with miR-128-3p and miR-376a-3p (*P<0.05). **(J, K)** Transwell assays were performed to assess the migration ability of HUVECs after transfection with miR-128-3p and miR-376a-3p (*P<0.05, ***P<0.001).

To discover abnormally regulated miRNAs that are secreted via serum-derived exosomes isolated from GC patients, we first collected serum from three GC patients and three healthy individuals to isolate exosomes for high-throughput sequencing. A total of six samples were tested, and the average amount of output data per sample was 25.012047 million reads. In total, 429 known miRNAs were identified, and 1932 novel miRNAs were predicted. Using the parameters of |log2-fold change|≥2.0 and *p*value < 0.05, we identified 1616 upregulated and 280 downregulated miRNAs (**Figure 2A**).

To further explore the pathways regulated by the differentially expressed miRNAs, we predicted the targets of the 30 most abundant miRNAs in exosomes using RNAhybrid, miRanda and TargetScan and then performed KEGG pathway analysis. The top 20 KEGG pathways were “PI3K-Akt signaling pathway,” “Rap1 signaling pathway,” “Toxoplasmosis,” “Thyroid hormone signaling pathway,” “Small cell lung cancer,” “RNA degradation,” “Regulation of actin cytoskeleton,” “Proteoglycans in cancer,” “Phagosome,” “Pathogenic Escherichia coli infection,” “Leukocyte transendothelial migration,” “Leishmaniasis,” “Human papillomavirus infection,” “Hematopoietic cell lineage,” “Focal adhesion,” “ECM-receptor interaction,” “Cell adhesion molecules (CAMs),” “Bacterial invasion of epithelial cells,” “Axon guidance,” and “Amoebiasis” (**Figure 2B**). Among the top 30 KEGG pathways, “PI3K-Akt signaling pathway” and “Rap1 signaling pathway” have been reported to be involved in angiogenesis.

### 3.3 MiR-128-3p is highly expressed in GC patient serum-derived exosomes

Since miRNAs with upregulated expression have more robust biomarker properties and more potential therapeutic targets in gastric cancer, we performed a literature review and prescreened five miRNAs with significantly increased expression (miR-28-3p, miR-128-3p, miR-660-5p, miR-144-5p, and miR-376a-3p) for functional validation (**Figure 2C**). After transfection with miR-128-3p and miR-376a-3p, the proliferation ability of AGS cells was significantly increased compared with that in the NC group (*P* < 0.05) (**Figure 2D**). Therefore, we selected miR-128-3p and miR-376a-3p for a further CCK8 assay (**Figures 2E, F**) and transwell assay (**Figures 2H–K**) in AGS cells and HUVECs for validation. Eventually, we focused on miR-128-3p because of its strong proliferation- and migration-promoting effects on both AGS cells and HUVECs. To confirm the abnormal regulation of miR-128-3p, we further measured its expression in exosomes isolated from the serum of 10 healthy individuals and 10 GC patients by qRT−PCR. The expression of miR-128-3p was significantly increased in serum exosomes isolated from the 10 GC patients compared to those from the 10 healthy individuals (**Figure 2G**).

### 3.4 GC-derived exosomes are internalized by HUVECs

**Figure 3:**
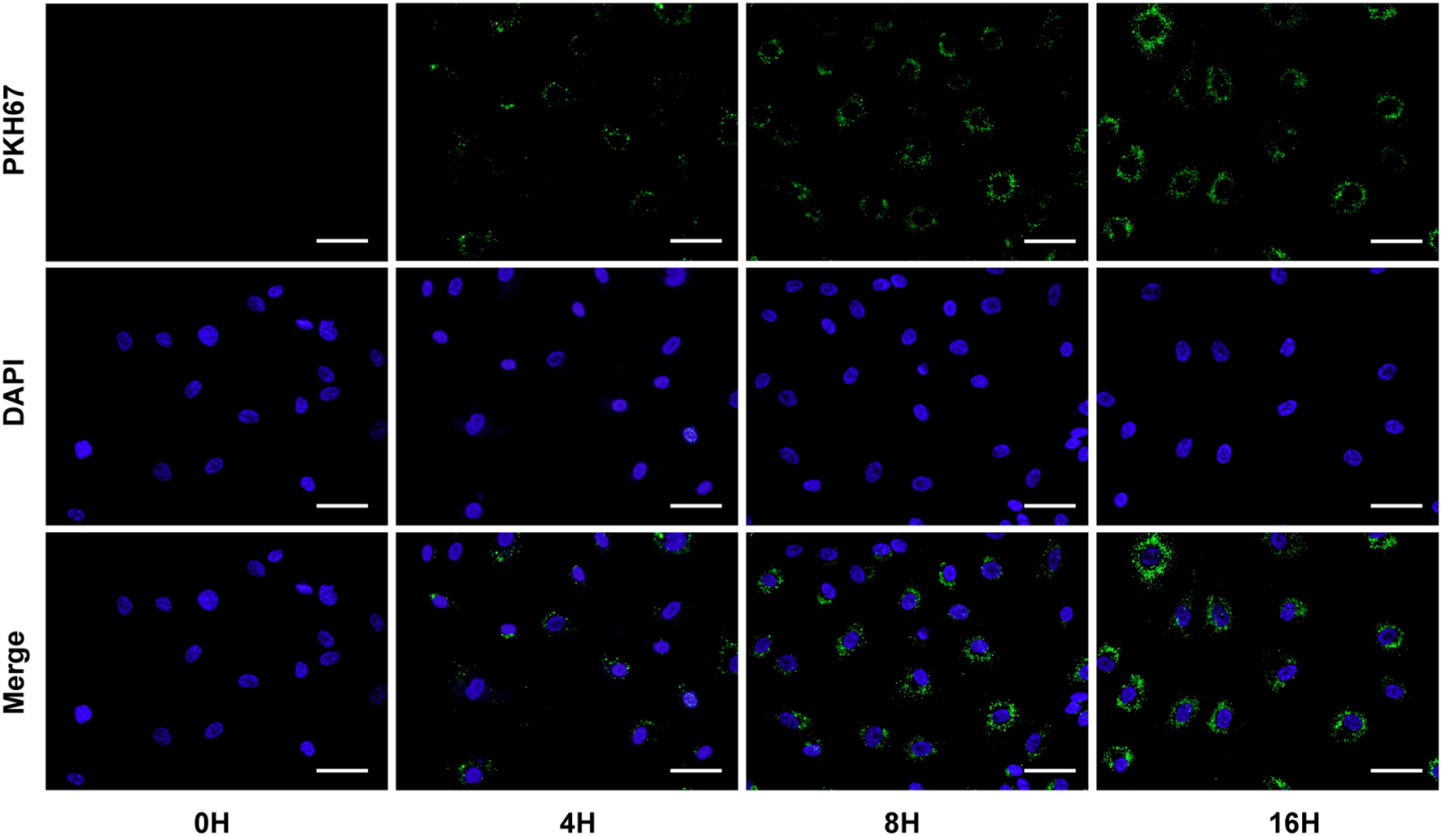
GC-derived exosomes were labelled with PKH67 green fluorescent dye and coincubated with HUVECs for different periods. Scale bar = 20 μm.

To determine whether exosomes secreted by gastric cancer cells can be delivered into endothelial cells, we cultured AGS cells and extracted exosomes from the culture supernatants. Then, these exosomes were labelled with PKH67 green fluorescent dye and incubated with HUVECs for different periods. The fluorescence micrographs showed that the internalized exosomes appeared as green fluorescent puncta around HUVEC nuclei in a time-dependent manner (**Figure 3**). The findings indicate that exosomes isolated from the supernatant of GC cells can enter endothelial cells and that the number of intracellular entry events increases with time.

### 3.5 GC exosomal miR-128-3p can promote HUVEC angiogenesis

**Figure 4:**
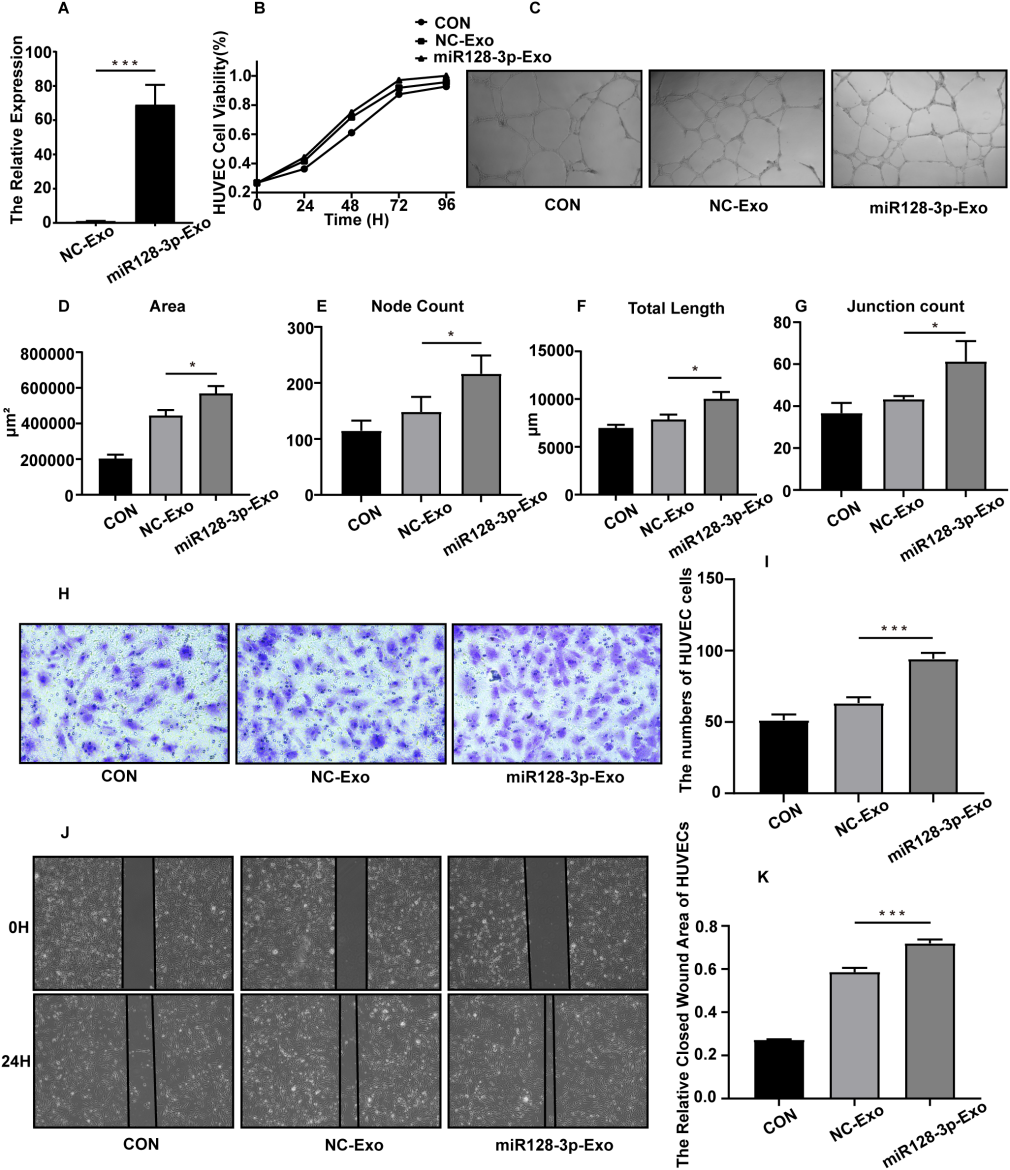
AGS exosomal miR-128-3p promotes HUVEC angiogenesis *in vitro*. The following assays were performed after HUVECs were treated for 24 h with exosomes (100 μg/mL) isolated from AGS cells. The control group was treated with the same volume of culture medium. **(A)** The overexpression efficiency of miR-128-3p in exosomes was verified (***P<0.001). **(B)** The proliferation ability of HUVECs was assessed using a CCK8 assay, and the proliferation curve was plotted. **(C–G)** A Matrigel tube formation assay was used to evaluate the tube formation ability of HUVECs (*P<0.05). **(H, I)** A transwell assay was used to assess the migration ability of HUVECs (***P<0.001). **(J, K)** A wound healing assay was used to assess the migration ability of HUVECs (***P<0.001).

To investigate the function of GC-derived exosomal miR-128-3p in angiogenesis, we first transduced the lentiviral vector overexpressing miR-128-3p or the NC vector into AGS cells. Next, two groups of exosomes were isolated from the supernatants of the above GC cells and named miR-128-3p-EXOs and NC-EXOs. We confirmed by qRT−PCR that the expression of miR-128-3p was significantly higher in miR-128-3p-EXOs than in NC-EXOs (**Figure 4A**). This finding indicated that we successfully overexpressed miR-128-3p in exosomes, and the function of exosomal miR-128-3p could thus be verified by evaluating the effects of these different groups of exosomes on HUVECs. After HUVECs were incubated with miR-128-3p-EXOs or NC-EXOs, with an equal volume of culture medium used as a control, we assessed the angiogenic ability of HUVECs by proliferation, Matrigel tube formation, transwell migration and wound healing assays. As expected, HUVECs incubated with miR-128-3p-EXOs showed increased proliferation (as assessed by the cell proliferation assay) (**Figure 4B**), increased tube formation (as assessed by the Matrigel tube formation assay) (**Figures 4C–G**), and increased migration (as assessed by the transwell migration assay and wound healing assay) (**Figures 4H–K**). These results indicate that GC exosomal miR-128-3p can promote HUVEC angiogenesis.

### 3.6 SASH1 is a direct target of miR-128-3p in HUVECs

**Figure 5:**
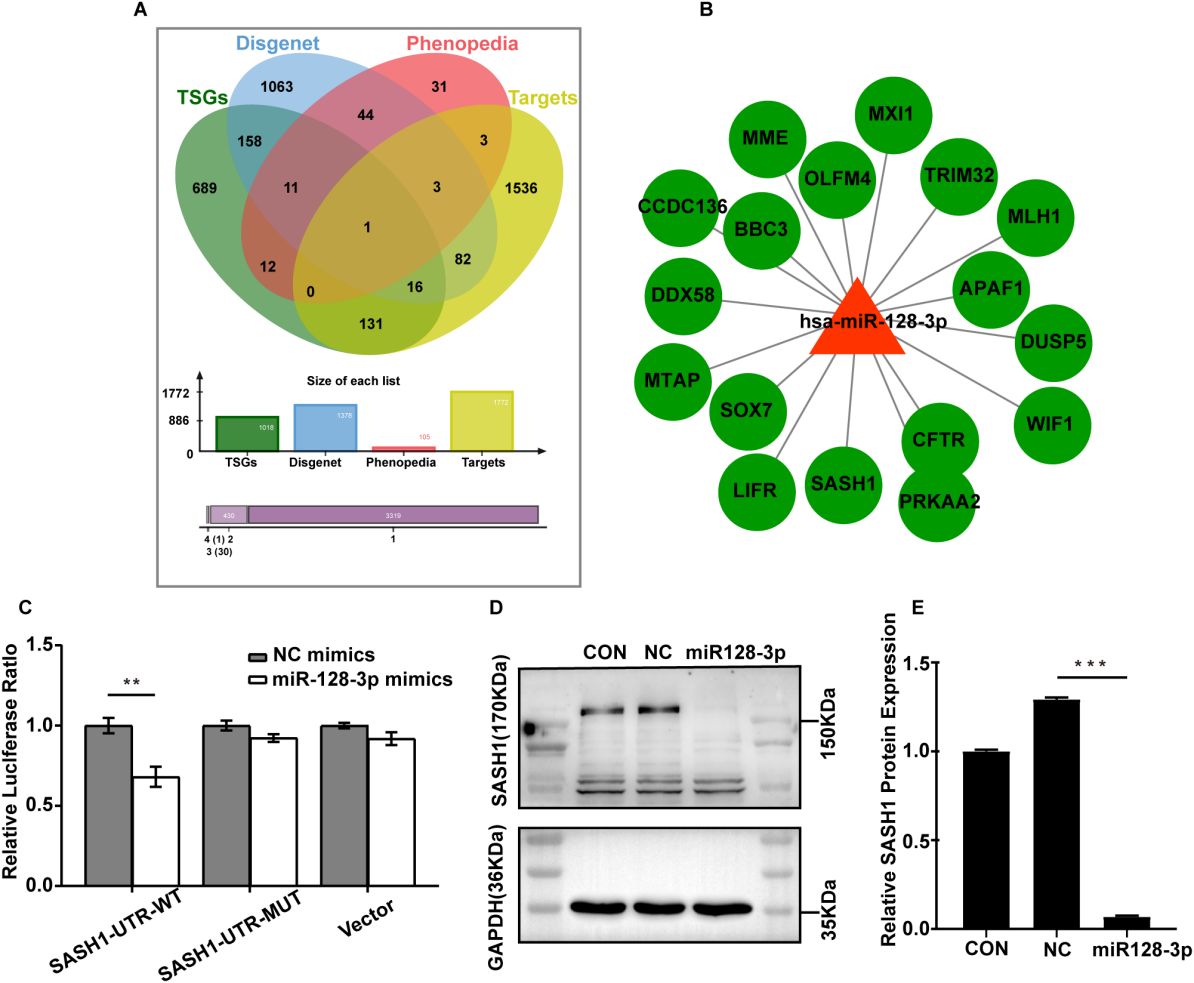
SASH1 is a direct target of miR-128-3p. **(A)** Venn diagram of tumour suppressor gene targets in gastric cancer predicted by four databases (miRWalk2.0, TSGene, DisGeNET and Phenopedia). **(B)** Regulatory network of hsa-miR-128-3p-regulated gastric cancer tumour suppressor target genes constructed by Cytoscape software. **(C)** HUVECs were cotransfected with the miR-128-3p mimic and the WT or MUT 3′ UTR construct. Luciferase activity was measured 48 h after transfection. Firefly luciferase activity was normalized to Renilla luciferase activity (**P<0.01). **(D, E)** Western blot analysis of SASH1 protein expression after transfection with the miR-128-3p mimic. GAPDH was used as a loading control(***P<0.001).

Since miRNAs inhibit target genes mainly at the posttranscriptional level, we were interested in tumour suppressor genes in gastric cancer that are regulated by miR-128-3p. To further understand the mechanism of GC exosomal miR-128-3p in regulating angiogenesis, we conducted bioinformatic analysis. The human gastric cancer oncogenesis-related gene targets were obtained by analysis of four databases (miRWalk2.0, TSGene, DisGeNET, and Phenopedia), and the Jveen tool was used to draw a Venn diagram (**Figure 5A**). A total of 17 oncogenic target genes were obtained by analysis of the DisGeNET database: SASH1, APAF1, CFTR, DUSP5, LIFR, MME, MTAP, MXI1, PRKAA2, OLFM4, WIF1, TRIM32, DDX58, BBC3, CCDC136, SOX7, and MLH1. We then constructed a regulatory network comprising these miR-128-3p-regulated oncogenic target genes in gastric cancer with Cytoscape software (**Figure 5B**). After combining these results with a review of the literature, we selected SASH1 for follow-up studies. To confirm the target relationship between miR-128-3p and SASH1, we performed a dual luciferase assay. The luciferase activity of the miR-128-3p mimic treatment group in HUVECs was significantly inhibited compared with that in the NC mimic-transfected group. In addition, when the predicted SASH1-3’ UTR site was mutated, the observed effect of miR-128-3p mimic treatment was abolished (**Figure 5C**). To further confirm the correlation between miR-128-3p and SASH1, we quantified the expression of SASH1 in HUVECs after transfection with the miR-128-3p mimic. Western blot analysis showed that the protein expression of SASH1 was significantly suppressed following transfection (**Figures 5D, E**). These results suggest that SASH1 expression is directly inhibited by miR-128-3p in HUVECs.

## 4 Discussion

Angiogenesis facilitates tumour development by providing essential oxygen and nutrients to tumour tissue and releasing metabolic waste products (8). Therefore, inhibition of neovascularization can inhibit the uptake of nutrients into the tumour, leading to a reduction in tumour progression. Recently, antiangiogenic therapy has shown remarkable results in a variety of cancers, such as gastric, lung, colorectal and breast cancers (9). However, single targeting and potential drug resistance may reduce the efficiency of antiangiogenic therapy. Therefore, studying the angiogenic processes involved in GC development is important for improving individualized treatment options for GC.

Exosomes, as novel mediators of intercellular information exchange, are able to transport various types of signalling molecules mediating intercellular signalling in the TME (13). Based on this role, exosomes not only are important components of the TME but also have the function of regulating the TME. It has been shown that exosomes can influence diverse cellular biological behaviours, including tumour angiogenesis, by affecting signalling communication processes in the TME (22), leading to changes in the metabolic state, proliferative capacity, invasive capacity and metastatic capacity of tumour cells (15, 23). In conclusion, exosomes are involved in influencing the TME and the various stages of tumour development and are closely associated with tumour growth, metastasis and recurrence.

All exosomes, regardless of their origin, are small round or spherical vesicles of 30-150 nm in diameter with surface expression of specific marker protein molecules such as HSP70, TSG101, Alix, SNARE, RAB GTPase and the four transmembrane proteins CD9, CD63 CD81, CD82 (12). Various methods are available for the isolation of exosomes, among which ultracentrifugation is the most commonly used. In addition, classical techniques such as density gradient centrifugation, immunoseparation, polymer-based precipitation and filtration have been applied to isolate exosomes. In this study, exosomes were isolated using the most commonly used method, ultracentrifugation, and identified by three different methods (transmission electron microscopy, nanoparticle tracking analysis and protein blotting). The results of these three identification methods confirmed that the characteristics of the isolated exosomes were consistent with the general characteristics of exosomes in terms of morphology, size and marker protein expression, which was sufficient to demonstrate the successful isolation of exosomes in our study.

Tumour-derived exosomes (TEXs) have become a popular topic in TME research, and TEX-mediated intercellular communication between tumour cells and endothelial cells plays an important role in tumour angiogenesis (24). TEXs can transfer specific bioactive molecules functioning as signalling molecules between tumour cells and endothelial cells, thus affecting endothelial cell function and in turn regulating the angiogenesis process (25). For example, A549 lung cancer cells can deliver miR-494 as a signalling factor to vascular endothelial cells through an exosome-mediated pathway, thereby promoting tumour angiogenesis by activating the Akt/eNOS pathway (26). Similarly, hepatocellular carcinoma cell-derived exosomes deliver miR-210 as a signalling molecule to endothelial cells, and miR-210 promotes tumour angiogenesis through two targets, SMAD4 and STAT6 (27). It is thus clear that TEX is an effective mediator of autocrine and paracrine communication between tumour cells and plays an important role in the biological process of tumour development. However, the mechanism by which exosomes enter receptor cells is not fully understood. Most studies have reported that exosomes release their cargo after internalization by the recipient cell (28, 29). In our study, fluorescently labelled exosomes were cocultured with HUVECs and visualized by microscopy, and the findings again showed that AGS cell-derived exosomes can be internalized by endothelial cells to perform their function.

In addition, exosomes contain source cell-specific metabolites and key functional molecules that reflect some of the biological characteristics of the cell of origin (30). There are significant differences in the rate of exosome release and the content of exosomes between normal and cancer cells. Furthermore, the unique molecular structure of exosomes protects their contents and allows them to remain highly stable in body fluids (11). Therefore, exosomal contents have the advantages of higher stability, greater specificity and better sensitivity than existing tumour markers.

In this study, we screened for the differentially expressed miRNAs in gastric cancer patient serum-derived exosomes by high-throughput sequencing and found by KEGG pathway analysis that some target genes of the differentially expressed miRNAs were involved in the regulation of angiogenesis-related signalling pathways, such as the PI3K-AKT and Rap1 pathways. After screening of the top-ranked differentially expressed miRNAs in combination with a literature review, we preselected five miRNAs (miR-128-3p, miR-144-5p, miR-660-5p, miR-28-3p and miR-376a-3p) that were differentially expressed in gastric cancer-derived exosomes. However, the mechanism of these miRNAs was not defined by this preliminary functional screen. MiR-128-3p, which exhibited proliferation- and migration-promoting effects on both AGS cells and HUVECs, was finally selected for follow-up experiments. Considering that gene sequencing of exosomal cargo can also have limitations, we collected a larger clinical sample and further validated the expression level of miR-128-3p in serum exosomes from GC patients and healthy controls using qRT−PCR. The results confirmed that miR-128-3p was abundantly enriched in gastric cancer-derived exosomes.

In previous studies, abnormal expression levels of miR-128-3p have been found in a variety of malignancies. For example, the miR-128-3p expression level was significantly decreased in tissues or cells from oesophageal squamous cell carcinoma (31), pancreatic cancer (32) and ovarian cancer (33) and significantly increased in tissues or cells from colorectal cancer (34), breast cancer (35) and osteosarcoma (36). In Currently, the potential functions and mechanisms of tumour exosomal miR-128-3p in tumour progression are gradually being explored. Jian Bai et al (34) found that colorectal cancer cell-derived exosomal miR-128-3p could directly inhibit its downstream target gene FOXO4, leading to activation of the TGF-β/SMAD and JAK/STAT3 signalling pathways to promote epithelial-mesenchymal transition in colorectal cancer and exert oncogenic effects.

To confirm the role of exosomal miR-128-3p in tumour angiogenesis during GC progression, we applied lentiviral transduction to stably overexpress miR-128-3p in AGS cells and verified the overexpression efficiency of miR-128-3p in isolated exosomes by qRT-PCR. The isolated exosomes were cocultured with HUVECs, and overexpression of miR-128-3p was found to significantly promote angiogenesis *in vitro*. We also identified SASH1, the target gene of miR-128-3p, through bioinformatic analysis and a literature review and further validated the target relationship between miR-128-3p and SASH1 by a dual luciferase assay and Western blot analysis.

The SASH1 gene was first identified as a tumour suppressor gene whose downregulation exerted oncogenic effects in breast cancer (37), and studies have continued to confirm that reduced SASH1 expression is strongly correlated with cell growth, proliferation and metastasis in a variety of malignancies (e.g., gastric (38), colorectal (39), and skin (40) malignancies). Another study showed an increase in endothelial cell apoptosis and upregulation of SASH1 expression following blockade of the VEGF receptor in human lung microvascular endothelial cells (41). In addition, Henri Weidmann et al (42) found that increases in proliferation, migration capacity and neovascularization were observed after silencing SASH1 in human aortic endothelial cells. In our study, we speculated that miR-128-3p may play a role in promoting angiogenesis of GC through the target of SASH1, and verified the targeting relationship between them by related experiments.

Based on the above theoretical basis, sequencing results and experimental results, we conducted a preliminary exploration of miRNAs within gastric cancer-derived exosomes and found that miR-128-3p was highly expressed in the serum exosomes isolated from GC patients. We further investigated the role and mechanism of miR-128-3p in gastric cancer-derived exosomes in angiogenesis and found that it facilitates angiogenesis. In addition, we found that SASH1 may be a target gene through which miR-128-3p functions. The results of our study have implications for the discovery of new gastric cancer biomarkers and the identification of new antiangiogenic therapeutic targets for GC. However, due to the small patient sample size in this study, further studies in a larger population are needed to determine the potential clinical value of our findings.

## 5 Conclusion

In this study, we used high-throughput sequencing to analyse serum exosomes isolated from gastric cancer patients and healthy individuals to explore differentially expressed miRNAs associated with gastric cancer. We found that miR-128-3p was highly expressed in serum exosomes from gastric cancer patients and further confirmed experimentally that gastric cancer cell-derived exosomal miR-128-3p could increase the angiogenic capacity of endothelial cells. In addition, this study found that SASH1 may be a target gene through which miR-128-3p functions, and the related mechanism needs further exploration. The above findings may identify new potential targets for the antiangiogenic treatment of gastric cancer.

## Data Availability

The original contributions presented in the study are included in the article/supplementary material. Further inquiries can be directed to the corresponding author/s.
